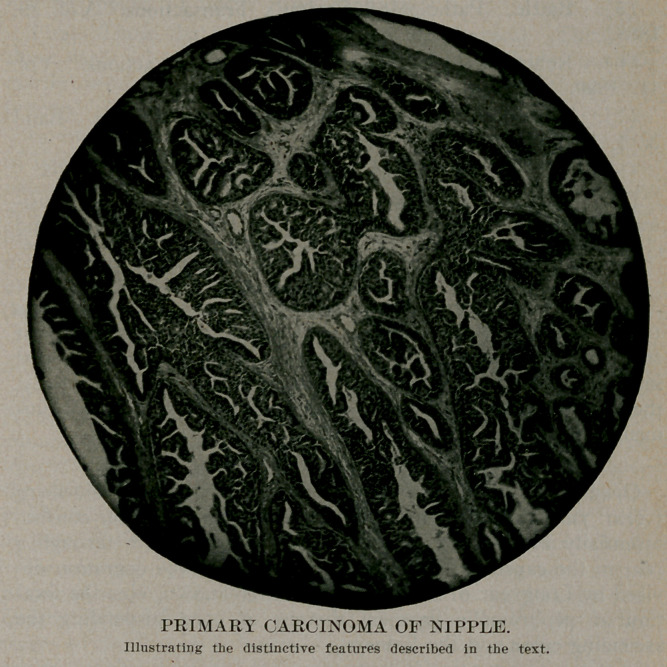# Primary Carcinoma of the Nipple

**Published:** 1913-04

**Authors:** Bernard F. Schreiner


### Primary Carcinoma of the Nipple.

**From Dr. Roswell Park's Surgical Clinic, Buffalo General Hospital**

By BERNARD F. SCHREINER, M.D.

ON account of the rarity of this condition, I thought it advisable to report a case that came under the observation of Dr. Park.

In a review of the literature the first cases reported were those by Sir Astley Cooper (1). He mentioned three cases of *fungus medullaris mammillae,* which occurred in men. He operated on all of them. One died of metastatic growth in the liver and the other two had no recurrence.

In 1846 Sir Benjamin Brodie (2) reported a case of carcinoma of the mammilla in a very fleshy woman, who had noticed a stony hardness in the nipple for a long time. This he treated by cauterization with chloride of zinc and caustic potash.

Rose (3), in 1846, also reported a case of scirrhous carcinoma of the mammilla, occuring in an elderly woman, who had noticed the tumor in the nipple for several years. The breast was removed. In this case the growth had spread to the mammary gland.

Prescott-Hewett (4), in 1872, reported a case of intracanalicular carcinoma of the mammilla, occuring in a woman of forty. This tumor was not only extirpated, but the specimen has been preserved, and is now in the museum of St. George's Hospital, No. 13A, Ser. 15. The microscopic diagnosis is cylindrical epithelial cell carcinoma, which sprang from and spread by way of the large and terminal milk ducts.

In 1888 Battles (5) reported a case of carcinoma of the ducts of the mammilla. This occurred in a woman of forty-five, mother of many children, all of whom she had nursed. For four months she had noticed a small lump the size of a hazel nut in the base of the right nipple. There was no auxiliary involvement.

In 1890 Robinson (6) reported a case of carcinoma of the ducts of the mammilla, occuring in a man who had consulted him on account of cancer of the tongue. During his examination of the patient he noticed that the left nipple was enlarged and uneven, and was surmounted by a small ulcer which was covered by a scab. He extirpated the tümor. On microscopical examination this proved to be a carcinoma which evidently sprang from the epithelial lining of the milk ducts.

Mandry (7), in 1893, reported a case of ulcerating epithelioma and carcinoma of the ducts of the mammilla. This occurred in a widow of sixty-one, mother of three children, all of whom she had nursed. For two years she had been aware of a small ulcer on the tip of the left nipple, which had begun to spread. The areola was infiltrated while the breast seemed normal. There were two palpable lymph nodes in the left axilla. The breast was removed. Microscopical examination revealed a carcinoma extending from the ulcerating surface down into the ducts.

Weil (8), in 1893, reported a case of ulcerated epithelioma and duct carcinoma of the mammilla. This occurred in a widow of forty-one, mother of six children, all of whom she had nursed. Four years previously she had had an abscess in the breast. During the past two years she had noticed a gradual enlargement of the left nipple. She also complained of some local pain. At the time of his examination the patient was poorly nourished, presenting a small tumor of the nipple, on the tip of which was a small ulcerating area with a depression in the middle. He removed the breast. Microscopical examination revealed numerous polygonal cells, which filled the alveoli and were surrounded by a dense connective tissue stroma.

The following is a brief history and the findings in the case herewith reported:

Miss Mary M., aged forty-two, American, clerk by occupation, entered the hospital September 9th, 1912. Complaint—enlargement of the left breast and nipple. Family and past history were good. There was no history of any injury. Menstruation began at the age of fourteen. For about one year the patient had noticed a few small lumps in the left breast. The nipple has always been prominent but is now very much larger, harder, and is quite tender upon pressure. There is no ulceration. The right breast also contains a few small nodules; its nipple, however, is normal. There are no palpable lymph nodes in either axilla.

On September 10, 1912, under ether, the left breast was removed by Dr. Park. The nodules in the breast proved on section to consist of multi-cystic dilatations of the ducts, though on microscopical examination there was no evidence of malignancy. The nipple was enlarged. A small tumor, about the size of a hazelnut and very hard, was found just beneath it. Microscopical examination of this tumor showed it to be a cylindrical cell carcinoma with alveolar structure. There were dilatations of some of the alveoli, which contained homogenous coagulated masses. The alveoli were separated by well marked and thickened connective tissue bands, which in places showed hyaline degeneration. The alveoli were lined by stratified cylindrical epithelium with a marked tendency to papillary ingrowth.. In a few alveoli the epithelium was arranged in a single layer, was of high columnar type, with deeply staining protoplasm and a basilar vesicular nucleus. In the stratified areas, especially where there was a tendency to papillary ingrowth, the boundary of the cells was difficult to determine. The neuclei were more solid and took a deep stain. Mitoses were rare. There were seen also in the preparation large ducts lined by stratified squamous epithelium, containing homogenous coagulated masses, such as were described above as being found in some of the alveoli. In certain areas could be found typical cylinderical cell carcinoma, apparently springing from the stratified squamous epithelium lining the ducts. The above mentioned so-called alveoli had moreover a distinct basement membrane.

PRIMARY CARCINOMA OF NIPPLE.

Illustrating the distinctive features described in the text.

In consideration of the above findings we believe we have in this specimen a cylindrical cell carcinoma, springing from the epithelium, lining the ducts of the nipple.

In conclusion I should like to say not only that primary carcinoma of the nipple is a very rare tumor, but that all other varieties are in this location extremely rare. Linfors (9), in 1900, collected twenty-seven cases of primary tumor of the nipple. Among those reported were myomata, fibromata, papillomata, epitheliomata, papillary cystomata and cystadenomata.

REFERENCES.

(1). Astley Cooper, Principles and Practice of Surgery. London, 1836, Tome II.

(2). Benj. Brodie, Lectures on Pathology and Surgery. London, 1846.

(3). Rose. Brodie Lectures on Pathology and Surgery. London, 1846.

(4). Prescott-Hewett, Shield, Diseases of the Breast, 1898.

(5). Battle. Pathological Society's Transactions. Vol. 39, 1888.

(6). Robinson. Pathological Society's Transactions. Vol. 41, 1890.

(7). Mandry. Bruns' Beiträge zur Klin. Chirurgie. Band X, 1, 1893.

(8). Weil. Prager Medic. Wochenschrift, No. 8, 1893.

(9). Linfors. Uber Primäre geschwülstbildung der Brustwarze und Warzenhofs. Monatschrift für Geburtshilfe und Gynekologie. 1900.

---

TECHNIC FOR REMOVAL OF FOREIGN BODIES.—H. Fründ, Bonn (*Zent. fur Chir.*, November 30, 1912). Fründ suggests the use of sterile needles used as finders before the fluoroscope, *i. e.*, thrust into the tissues so as to come in contact with the foreign body, there to remain until the foreign body is removed, incision immediately following.

---

OPERATION FOR MOBILE KIDNEY.—A. Narath, Heidelberg (*Zent. fur Chir.*, November 30, 1912). The operation consists essentially in impaling the kidney on the twelfth rib through a rent in the capsule. It is to be regretted that the operation devised by Longyear of Detroit is not better known, viz., the fixation of nephro-colic ligament thereby at once suspending the ascending color and the kidney.

---

NEOSALVARSAN.—*International Journal of Surgery*, September, 1912. Editorial quotation: When . . . . . neosalvarsan is suspended in pure glycerine and then dissolved by the addition of distilled water . . . we have an intramuscular injection that is practically painless, and in the majority of cases absolutely so.

## Figures and Tables

**Figure f1:**